# Modification of CoFe Prussian Blue Structure by N_2_ Plasma for Enhanced Electrocatalysis

**DOI:** 10.3390/ma19081580

**Published:** 2026-04-15

**Authors:** Jiaming Zhao, Guangrui Zhang, Lele Gao, Jing Zhao, Yuanbo Wang, Guoling Li

**Affiliations:** 1 Institute of Materials for Energy and Environment, College of Materials Science and Engineering, Qingdao University, Qingdao 266071, China; w3115815204@163.com (J.Z.); wangyuanbo@qdu.edu.cn (Y.W.); 2 Rare Earth Advanced Materials Technology Innovation Center, Inner Mongolia Northern Rare Earth Advanced Materials Technology Innovation Co., Ltd., Baotou 014030, China; zhangguangrui@reamtic.cn (G.Z.); gaolele@reamtic.cn (L.G.); zhaojing@reamtic.cn (J.Z.); 3 Key Laboratory of Advanced Energy Materials Chemistry (Ministry of Education), Nankai University, Tianjin 300071, China

**Keywords:** N_2_ plasma, CoFe Prussian blue analog, nitrogen doping, vacancies, electrocatalysis

## Abstract

The efficiency of hydrogen production via water electrolysis is severely constrained by the sluggish reaction kinetics of the oxygen evolution reaction (OER). Herein, we constructed a nitrogen-doped CoFe Prussian blue analog (CoFePBA-N) electrocatalyst with a nanosheet-assembled cubic architecture by plasma. Plasma treatment induces morphological reconstruction and introduces nitrogen dopants and abundant vacancies, which not only increase the number of exposed active sites but also modulate the electronic structure of Co/Fe centers. Consequently, the optimized CoFePBA-N catalyst achieves a current density of 500 mA cm^−2^ at low overpotentials of 322, 344, and 374 mV in alkaline freshwater, alkaline simulated seawater, and alkaline natural seawater, respectively. Furthermore, the catalyst maintains stable operation for over 300 h in alkaline freshwater and nearly 270 h in alkaline natural seawater, exhibiting exceptional durability. The enhanced catalytic performance is attributed to the synergistic effects of nitrogen doping, vacancies, and improved charge-transfer capability. This study provides an effective approach for modulating the electronic structure of Prussian blue analogs, thereby enabling efficient alkaline water and seawater electrolysis.

## 1. Introduction

During the electrolysis of water, the reaction rate of the four-electron oxygen evolution reaction (OER) is extremely slow, which is required for hydrogen production, remains a major obstacle to its widespread application. To accelerate the OER rate and ensure operational stability, the development of electrocatalysts that are not only efficient and durable but also low in cost is urgently required. Among the various candidate materials, Prussian blue analogs (PBAs)—a class of cyanide-bridged transition-metal coordination polymers—have emerged as promising electrocatalysts for water splitting, owing to their open framework structures and uniformly distributed metal components. However, pristine PBAs generally suffer from poor electrical conductivity and insufficient active sites, which limit their practical applications. To address these challenges, heteroatom doping through electronic structure regulation and a microstructural project has been proposed as an effective strategy to optimize catalytic performance.

Nitrogen (N) is considered a particularly promising dopant because of its strong electronegativity and electron-withdrawing capability, which can induce charge redistribution and create abundant catalytic active sites. Nitrogen doping can not only regulate the electronic configuration of metal active centers to intensify their intrinsic activity, but also improve the electroconductibility and stability of the catalyst. For instance, Yi et al. successfully fabricated a hybrid-metal CoFePBA material with a hierarchical one-dimensional/three-dimensional string-bead architecture and subsequently obtained a highly active S-CoFePBA/NF through in situ conversion and sulfidation processes. The optimized S-CoFePBA exhibited excellent trifunctional electrocatalytic performance [1]. In another study, Yuan et al. developed a simple strategy to engineer cobalt vacancies through a process involving a CoFePBA precursor, phosphating-heating decomposition, tannic acid etching, and subsequent phosphorization [2]. By controlling the concentration of tannic acid, the density of cobalt vacancies could be effectively tuned. This study developed a simple method for creating metal cation vacancy defects and provided mechanistic insights for the exploration of multi-vacancy heterogeneous structure catalysts for efficient alkaline water electrolysis. CoFePBA possesses a typical cubic open structure, in which cobalt and iron metal ions are bridged by cyano groups to form a stable and highly ordered three-dimensional network. This porous structure can not only display a large number of high-density active sites but also provide sufficient electrolyte transport channels and charge transfer, enabling rapid electron transfer and ion transport. Šljukić et al. prepared cobalt-iron nanoparticle catalysts using three-dimensional carbon aerogel as the carrier and investigated their application in electrochemical energy conversion and storage systems. They optimized the structure and catalytic performance of the catalysts by using microwave irradiation in one-step and two-step methods. The electrochemical results showed that the material had good charge storage performance in an alkaline medium. The studied material mainly stored charges through the pseudo-Faraday mechanism, and the synthesized Co-Fe/CA-1 became a promising material for energy storage and electrocatalytic processes [3]. Li et al. suggested that rational interface engineering and morphology control are regarded as effective strategies to modulate the electronic structure and enhance the activity of spinel materials [4]. By using an in situ assembly method, CoFe_2_O_4_-coated CoFe alloy nanoparticles were prepared, which effectively improved the electrical conductivity of the catalyst particles. The as-prepared CoFe/CoFe_2_O_4_@NC catalyst exhibited favorable oxygen evolution catalytic performance. The formation of the CoFe/CoFe_2_O_4_ interface strengthened the adsorption capacity of the catalyst toward OER intermediates and reduced the energy barrier of the rate-determining step during the reaction, thereby promoting the OER kinetics. Jiao et al. designed a ZIF-derived electrocatalyst Co@Fe-P with a core–shell structure using a Co-based compound as the core and an Fe-based compound as the shell, connected by Co-O-Fe and Fe-O-P bonds [5]. The electrocatalyst exhibits enhanced OER performance with the advantages of low overpotential and a low Tafel slope. It greatly promotes electron transfer from the CoOOH core to the FeOOH shell, which provides insights for designing efficient electrocatalysts for the oxygen evolution reaction. Benefiting from these advantages, CoFePBA can serve as an excellent electrocatalytic precursor and active material to address key issues in the electrocatalytic process, such as poor conductivity, insufficient active sites, easy structural collapse, and unreasonable intermediate adsorption. However, it is necessary to explore how to achieve multifunctional integration while retaining this favorable structural framework. Room-temperature plasma is an intermediate surface modification technology with low thermal budget and high chemical reactivity, in which the crystal structure and favorable morphology of the precursor can be well preserved [6,7,8].

Inspired by these advancements, the combination of nitrogen doping and vacancy engineering has provided a highly promising approach for further optimizing the electrocatalytic performance of PBAs. Notably, the ionic radii of iron and cobalt are similar, rendering them mutually compatible and enabling the co-formation of high-valence metal active sites. These sites exhibit remarkable intrinsic activity toward the alkaline oxygen evolution reaction. Based on these insights, in this work, the plasma-enhanced chemical vapor deposition (PECVD) nitridation strategy was employed to transform solid cubic CoFePBA, which was in situ grown on nickel foam (NF), into nitrogen-doped nanosheet-assembled cubic CoFePBA. The introduced nitrogen atoms regulate the electronic distribution around Co and Fe, promoting electron transfer from Fe to Co and activating the metal centers for catalysis. Introducing vacant sites can construct unsaturated coordination active sites, precisely controlling the microscopic electronic structure and adsorption energy of the intermediate molecules. Finally, CoFePBA-N not only exhibits excellent catalytic ability in fresh water environments, but also maintains high catalytic activity in seawater while maintaining long-term stability.

## 2. Results and Discussion

### 2.1. Preparation and Characterization

**Figure 1:**
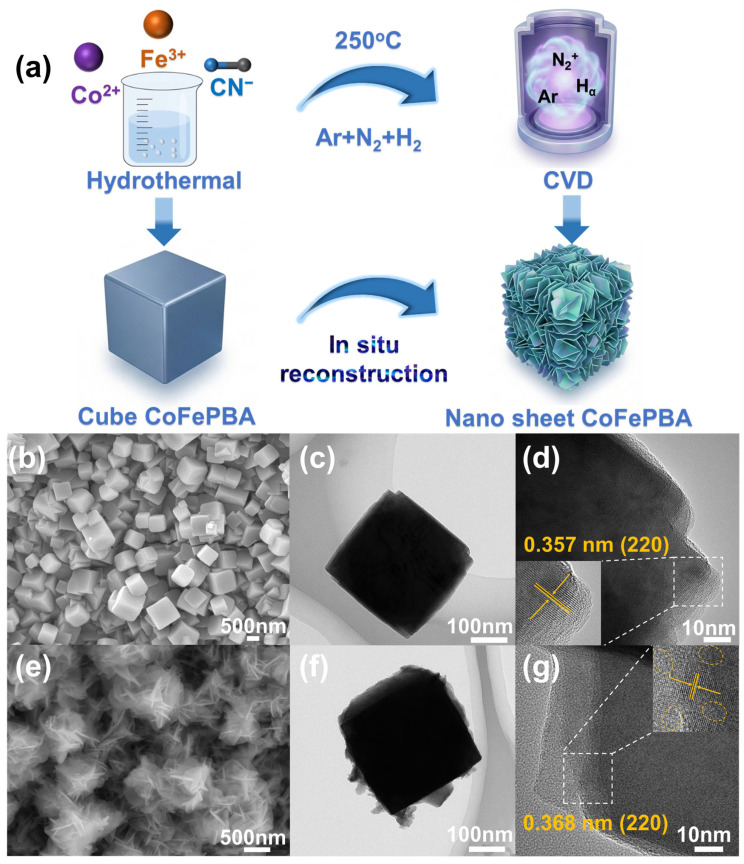
The preparation process of CoFePBA-N (**a**), SEM images of CoFePBA (**b**), and CoFePBA-N (**e**). TEM images and HR-TEM images of CoFePBA (**c**,**d**) and CoFePBA-N (The yellow circle in the enlarged image represents lattice distortion) (**f**,**g**).

The synthesis route of CoFePBA adopted in this paper is shown in Figure 1a. First, CoFePBA was directly uniformly grown on nickel foam, after which the obtained material was placed in a CVD system and subjected to plasma treatment under a mixed atmosphere of Ar, H_2_, and N_2_. The different states of nickel foam involved in this study are presented in Figure S1. Figure 1b shows the scanning electron microscope (SEM) image of the sample, from which it can be seen that the as-synthesized CoFePBA exhibits smooth cubic structures. After plasma treatment, the obtained CoFePBA-N evolves into a cube-like architecture composed of interconnected nanosheets (Figure 1e). The microscopic morphology of CoFePBA (Figure 1c) and CoFePBA-N (Figure 1f) can be observed in the transmission electron microscope (TEM) images. In the high-resolution transmission electron microscope images, the lattice spacing of the (220) crystal plane in CoFePBA is 0.357 nm (Figure 1d), while in CoFePBA-N, the lattice spacing of the same crystal plane is 0.368 nm (Figure 1g). At the same time, in the TEM magnification image, obvious deformation features can be clearly observed, clearly indicating the lattice deformation caused by nitrogen doping and vacancies. Such a structure could remarkably increase the surface area and expose numerous active sites, thereby facilitating rapid ion diffusion during the catalytic process. Furthermore, the elemental distribution spectra also reveal that the plasma-treated CoFePBA-N exhibits a higher nitrogen content, with uniform distribution (Figure S2a–c). This process enabled the uniform incorporation of nitrogen into the material, resulting in N-doped CoFePBA.

**Figure 2:**
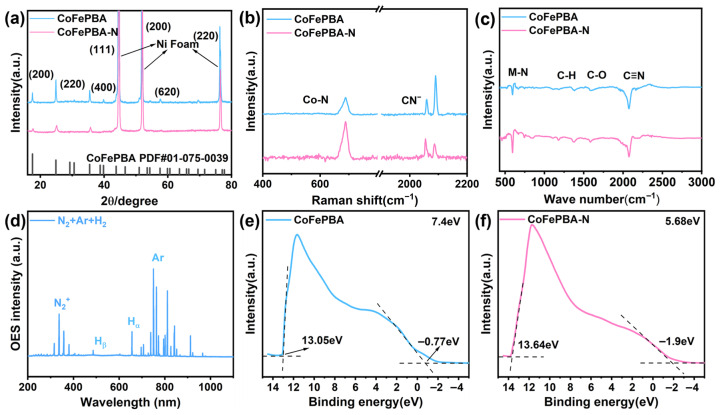
XRD (**a**) and Raman spectra (**b**) of CoFePBA and CoFePBA-N infrared spectra (**c**), OES spectrum (**d**), and UPS ultraviolet spectra of CoFePBA (**e**) and CoFePBA-N (**f**) (The dotted line represents the tangent of the curve.).

The crystal structure was tested by using X-ray diffraction (XRD). As shown in Figure 2a, the characteristic peaks of CoFePBA, located at 17°, 25°, 35°, and 58°, can be assigned to the (200), (220), (400), and (620) crystal planes, respectively, corresponding to the characteristic peak positions of the CoFePBA standard card (PDF#01-075-0039) [9,10]. The three distinct characteristic peaks at 44°, 51°, and 76°, respectively, belong to the (111), (200), and (220) planes of Ni Foam. After plasma treatment, the characteristic diffraction peaks of CoFePBA are still observable, however, their intensities decrease significantly. This situation may be attributed to the structural reconstruction induced by the bombardment of high-energy plasma. Through Raman spectroscopy analysis, the structural characteristics of CoFePBA-N were further explored. As displayed in Figure 2b, the peak at 688 cm^−1^ can be attributed to the M-N stretching mode in CoFePBA, while the peaks at 2056 cm^−1^ and 2085 cm^−1^ correspond to the vibration modes of the cyano groups in CoFePBA [11,12]. After plasma treatment, the content of the cyano group in CoFePBA-N decreased significantly. The result indicates a partial breakage of the cyano group and the formation of vacancy defects, and the intensity of the M-N characteristic peak in the Raman spectrum increases significantly, indicating that more metal–nitrogen bonds are formed in the material after plasma treatment. Fourier transform infrared (FTIR) spectroscopy was further performed to investigate the bonding characteristics of the materials. As shown in Figure 2c, the characteristic peak observed at 593 cm^−1^ can be attributed to the formation of metal–nitrogen (M-N) bonds. The peaks at 1382 cm^−1^ and 1583 cm^−1^ in CoFePBA are ascribed to C-H and C-O vibrations, respectively, while the peak at 2075 cm^−1^ corresponds to the stretching vibration of cyano groups [13,14]. After plasma treatment, the intensity of the characteristic peak related to M-N bonds also increases significantly, demonstrating that plasma treatment enhances the content of metal–nitrogen bonds in CoFePBA-N. Both the Raman and FTIR results collectively demonstrate that nitrogen has been successfully and uniformly incorporated into the material. To further identify the plasma treatment process, the in situ optical emission spectroscopy (OES) was employed to observe the types of active plasma (Figure 2d). In the Ar-H_2_-N_2_ plasma spectrum, a distinct characteristic peak at a wavelength of 337.02 nm could be observed, which is attributed to the N_2_^+^ radical species. The presence of N_2_^+^ radicals is a key factor that enables nitrogen doping under low-temperature conditions. Furthermore, the characteristic peak at 656.27 nm can be attributed to the Hα emission line, while the signal at 750.08 nm corresponds to the characteristic emission of Ar [15]. To further investigate the intrinsic electronic interactions of these materials, ultraviolet photoelectron spectroscopy (UPS) technology was employed for characterization. In Figure 2e,f, by using $\Phi =E_{He1}-(E_{cutoff}-E_{onset})$, the WKs (Work function) for CoFePBA and CoFePBA-N were determined to be 7.40 eV and 5.68 eV, respectively. A lower work function gives rise to enhanced electron mobility within the metal orbitals [16]. The change in work function mediated by electronic coupling thus promotes surface charge transfer to the intermediates, ultimately enhancing the electrocatalytic performance.

**Figure 3:**
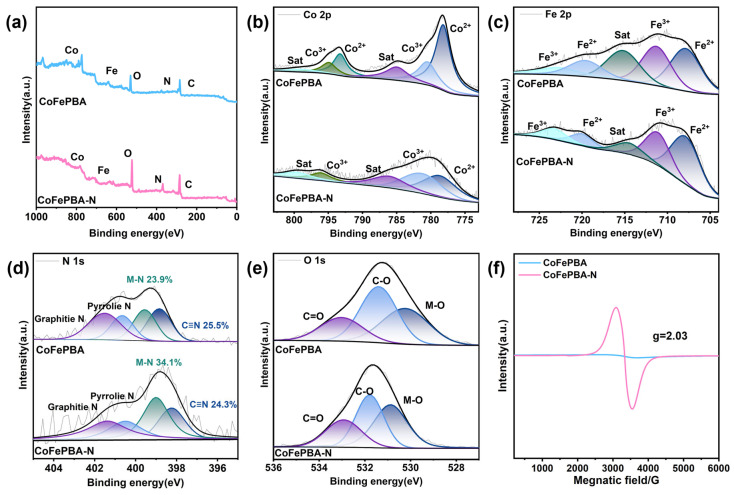
High-resolution XPS spectra of CoFePBA and CoFePBA-N. (**a**) Overall survey of pristine, (**b**) Co 2p, (**c**) Fe 2p, (**d**) N 1s, (**e**) O 1s. (**f**) EPR spectra of CoFePBA and CoFePBA-N.

To clarify the mechanism of the influence of nitrogen doping on the electronic structure, X-ray photoelectron spectroscopy (XPS) technology was employed to conduct systematic tests on CoFePBA and CoFePBA-N. The survey spectra reveal the presence of Co, Fe, O, N, and C elements in the materials (Figure 3a). The spectral features located at 778.2 and 793.2 eV (Figure 3b) are characteristic of Co^2+^ species [17]. Compared with CoFePBA, the characteristic peaks corresponding to CoFePBA-N show a more significant positive shift in binding energy. In the Fe 2p spectrum, the peaks at 707.9 eV and 711.3 eV of CoFePBA can be attributed to the Fe^2+^ and Fe^3+^ species, respectively (Figure 3c) [18]. After plasma treatment, the Fe 2p peaks of CoFePBA-N also display a positive shift in binding energy. In the N 1s spectrum (Figure 3d) of CoFePBA, the peak at 398.8 eV is assigned to C≡N, the peak at 399.5 eV corresponds to M-N bonds, the peak at 400.7 eV belongs to Pyrrolic N, and the peak at 401.5 eV is attributed to Graphitic N [19,20]. However, after plasma treatment, the N 1s spectrum of CoFePBA-N exhibits a low binding energy shift. Furthermore, the calculation results from peak area calculus indicate that N_2_ plasma increased the proportion of metal–nitrogen bonds from 23.9% to 34.1%, and the proportion of the C≡N bond decreased from 25.5% to 24.3%. This further confirms the successful incorporation of nitrogen atoms and the reduction in C≡N bonds. Meanwhile, the O 1s spectra also show a positive binding energy shift (Figure 3e), whereas the C 1s spectra display no significant change (Figure S3). The positive shift in the spectral peaks suggests that the strong electronic coupling induced by nitrogen introduction effectively regulates the electronic structure of CoFePBA-N. In order to measure the specific changes in C≡N, electron paramagnetic resonance (EPR) spectroscopy was further employed to evaluate the formation of defects. As shown in Figure 3f, g = 2.03 is confirmed to be the characteristic position of V_CN_. Compared with the pristine CoFePBA, the plasma-treated CoFePBA-N exhibits a significantly enhanced EPR signal, indicating the concentration of vacancy-type defects shows a significant increase trend [21]. This type of vacancy can promote the exposure of metal active sites and optimize their local coordination environment, thereby increasing the number of accessible metal catalytic centers that can be rapidly electrochemically reconstructed into the active CoOOH phase [22,23,24]. Consequently, the defect-rich CoFePBA-N is expected to exhibit superior pre-catalytic activation behavior and enhanced OER activity.

### 2.2. OER Performance in Alkaline Electrolyte

**Figure 4:**
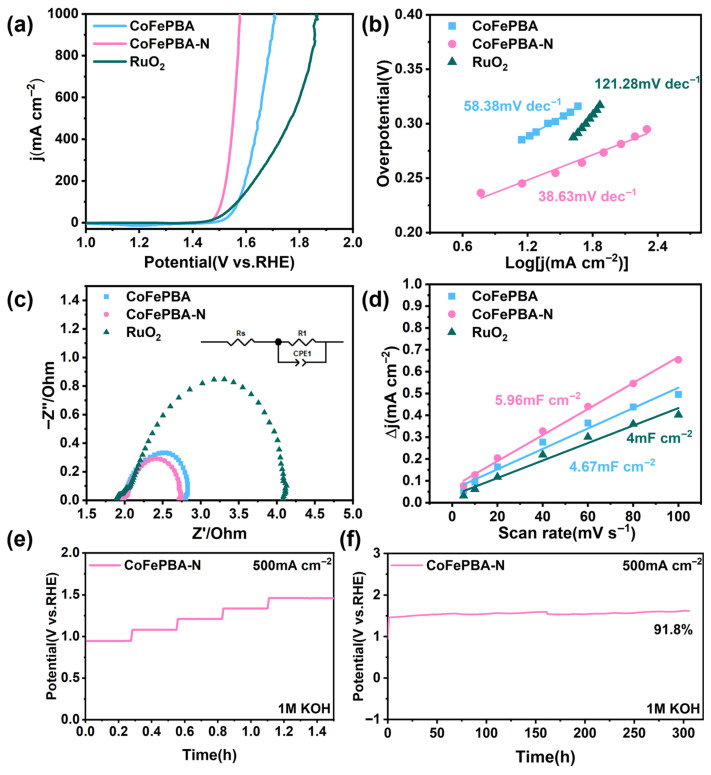
The electrocatalytic oxygen reduction reaction performance in a 1.0 M KOH. (**a**) Polarization curve, (**b**) Tafel slope, (**c**) EIS Nyquist plots, and (**d**) C_dl_ values of CoFePBA-N, CoFePBA, and RuO_2_. (**e**,**f**) Potentiostatic stability test with iR compensation of CoFePBA-N at 500 mA cm^−2^ in 1.0 M KOH.

At room temperature, using a standard three-electrode system and a scanning rate of 5 mV s^−1^, the electrocatalytic oxygen evolution reaction performance of the material was tested by linear sweep voltammetry (LSV). Firstly, its catalytic activity was evaluated in 1 M KOH. In the field of electrocatalysis research, RuO_2_ is recognized as a commercial benchmark catalyst for the oxygen evolution reaction and is often used as a reference material for performance comparison. As shown in Figure 4a, CoFePBA requires an overpotential of 420 mV to reach 500 mA cm^−2^ in alkaline freshwater, which is comparable to that of RuO_2_ (531 mV). In contrast, CoFePBA-N exhibits a much lower overpotential of only 322 mV under the same conditions. Furthermore, the Tafel slope of CoFePBA is 58.38 mV dec^−1^, which is significantly smaller than that of RuO_2_ (178.09 mV dec^−1^), while CoFePBA-N demonstrates the lowermost tafel slope of 38.63 mV dec^−1^ (Figure 4b). These results indicate that CoFePBA-N possesses superior OER kinetics compared with CoFePBA and RuO_2_, particularly at high current densities. The Nyquist plot (Figure 4c) obtained from the electrochemical impedance spectroscopy (EIS) test indicates that the charge-transfer resistance, from smallest to largest, is CoFePBA-N < CoFePBA < RuO_2_, further demonstrating the enhanced charge-transfer capability of CoFePBA-N. We recorded the cyclic voltammogram curves under different scanning rates and determined the corresponding electrochemical double-layer capacitance (C_dl_) values in 1 M KOH. The results are shown in Figure 4d and Figure S4, respectively. The C_dl_ value of CoFePBA-N (5.96 mF cm^−2^) is higher than that of CoFePBA (4.67 mF cm^−2^) and RuO_2_ (4 mF cm^−2^), indicating that the nanosheet-assembled structure of CoFePBA-N exposes more electrochemically active sites. Furthermore, we performed multiple measurements on CoFePBA-N to eliminate the influence of randomness, and the multiple measurements showed that it still maintains nearly consistent electrochemical performance, indicating its reproducibility (Figure S5). To avoid the influence of the electrochemical active surface area (ECSA) on the OER activity, we conducted a further analysis of the LSV curves that were standardized with respect to ECSA (Figure S6) [3,25,26]. Moreover, the normalized LSV curves calculated from the turnover frequency further indicate that CoFePBA-N still exhibits outstanding intrinsic catalytic activity (Figure S7). CoFePBA-N still exhibits superior catalytic activity after normalization, suggesting that its splendid OER performance originates not only from the enlarged active surface area but also from its intrinsically improved catalytic properties. Compared with recently reported data, the OER performance delivered by CoFePBA-N exhibits distinct advantages (Table S1). Long-term stability is another key indicator for evaluating electrocatalysts. As shown in Figure 4e,f, under an electric current density of 500 mA cm^−2^, after 300 h of testing, the OER performance of CoFePBA-N showed almost no degradation and still maintained an actual activity percentage of 91.8%. This further proves that this catalyst has excellent catalytic activity and stability in 1 M KOH.

**Figure 5:**
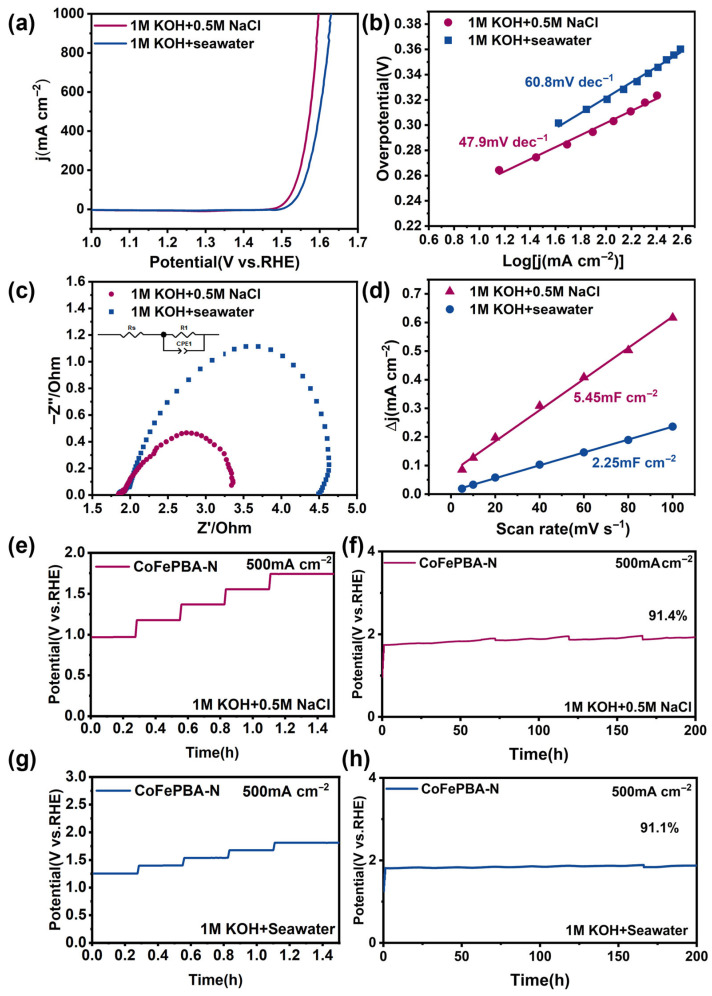
(**a**) Polarization curves, (**b**) Tafel slopes, (**c**) EIS Nyquist plots, and (**d**) C_dl_ of CoFePBA-N in different electrolyte solutions. (**e**,**f**) Potentiostatic stability test with iR compensation of CoFePBA-N at 500 mA cm^−2^ in 1.0 M KOH + 0.5 M NaCl and (**g**,**h**) 1.0 M KOH + Seawater.

Based on its outstanding OER performance in freshwater, the catalytic activity of CoFePBA-N was further examined in seawater systems. As shown in Figure 5a, the OER activity of CoFePBA-N was evaluated in alkaline simulated seawater (1 M KOH + 0.5 M NaCl) and natural seawater (1 M KOH + Seawater). In simulated seawater, the catalyst requires an overpotential of 344 mV at 500 mA cm^−2^, which is close to its performance in freshwater. In addition, the Tafel slope (47.9 mV dec^−1^, Figure 5b), EIS results (Figure 5c), and C_dl_ (5.45 mF cm^−2^, Figure 5d) are comparable to those obtained in freshwater; therefore, it can be seen that chloride ions have no significant effect on the reaction rate during the electrolysis process or on the exposure state of the active sites. Furthermore, the performance of the catalyst in natural seawater was also tested in detail. As shown in Figure 5a, the overpotential is only 374 mV at a current density of 500 mA cm^−2^. We also tested the cyclic voltammetry (CV) curves at different scan rates (Figure S8). Notably, the overpotential required to trigger the chlorine evolution reaction (2Cl^−^ + 2OH^−^→OCl^−^ + H_2_O + e^−^) is generally higher than 480 mV. To determine whether active chlorine species were generated during OER, the electrolyte after the reaction was analyzed. If active chloride ions (OCl^−^) exist in the system, these ions will react with 0.5 M potassium iodide (KI) and cause a change in the solution’s color [27]. As shown in Figure S9, after the addition of potassium iodide, no color change was observed in either the alkaline simulated seawater or natural seawater systems. This result indicates that CoFePBA-N can effectively suppress chlorine evolution and is suitable for seawater electrolysis. In natural seawater, the Tafel slope is 60.8 mV dec^−1^ (Figure 5b), the Nyquist plot obtained in natural seawater (Figure 5c) exhibits a larger impedance arc compared with other electrolytes, and the C_dl_ value is 2.25 mF cm^−2^ (Figure 5d). Although in natural seawater, the activity of the OER is slightly lower than that observed in simulated seawater, it still represents a promising result. The slight decrease in catalytic activity is mainly attributed to the highly complex composition of natural seawater. The cations, such as Ca^2+^ and Mg^2+^, present in natural seawater, tend to adsorb onto the electrode surface, partially blocking the active sites, which leads to a slight reduction in catalytic performance [27,28]. Under long-term high current density operation conditions, the durability of the catalyst is especially important. As shown in Figure 5e–h, CoFePBA-N can maintain stable operation for up to 200 h in simulated seawater and natural seawater at a current density of 500 mA cm^−2^. Additionally, the catalysts, respectively, retained 91.4% and 91.1% of the actual retention percentage.

### 2.3. Practical Performance in Overall Water Splitting

**Figure 6:**
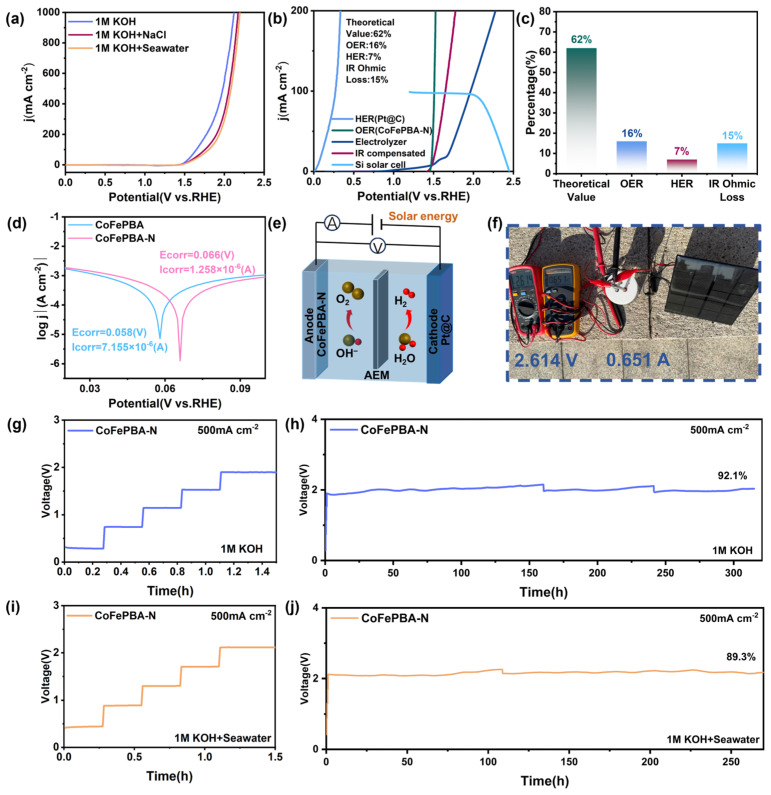
Performance of CoFePBA-N||Pt@C overall water-splitting tests in different electrolytes. (**a**) LSV curves. (**b**,**c**) Solar-driven performance in alkaline freshwater. (**d**) Corrosion polarization curves in alkaline seawater. (**e**) Circuit diagram of the simulation device. (**f**) Photograph of the sunlight-powered overall water-splitting device. Stability tests of overall water splitting in alkaline freshwater (**g**,**h**) and seawater (**i**,**j**).

To evaluate the practical application value of CoFePBA-N, we set up a dual-electrode electrolytic cell consisting of CoFePBA-N||Pt@C, with CoFePBA-N as the anode and Pt@C as the cathode, to achieve the overall decomposition of water. The catalytic performance of overall water electrolysis was examined in different electrolytes. As shown in Figure 6a, the CoFePBA-N||Pt@C electrolyzer requires cell voltages of 1.98 V, 2.05 V, and 2.08 V to achieve a current density of 500 mA cm^−2^ in alkaline freshwater, simulated seawater, and alkaline natural seawater, respectively. The actual overall electrolysis efficiency can be directly derived from the LSV data presented in Figure 6b,c. In alkaline freshwater, the practical overall electrolysis efficiency reaches as high as 62%, where the energy loss contributions are quantified as 16% from oxygen evolution reaction polarization, 7% from hydrogen evolution reaction polarization, and 15% from ohmic losses. Considering its promising performance in seawater, the corrosion resistance of the catalyst was further systematically investigated. As shown in Figure 6d, the erosion polarization curves measured in natural seawater demonstrate that compared with the original CoFePBA, it can be seen that CoFePBA-N has a more positive erosion potential and a lower erosion current density, confirming its enhanced corrosion resistance [29]. According to the circuit design shown in Figure 6e, the electrolysis system was operated under natural sunlight irradiation. As illustrated in Figure 6f and the accompanying video (Video S1), a large number of microbubbles rapidly form and continuously detach from the electrode surface, confirming the efficient solar-driven photoelectrochemical seawater splitting process. To evaluate the long-term operational stability for efficient water splitting, chronopotentiometry measurements were conducted for the CoFePBA-N||Pt@C electrolyzer at a current density of 500 mA cm^−2^. As shown in Figure 6g,h, the two-electrode system exhibits excellent stability, maintaining operation for more than 300 h at 500 mA cm^−2^ with only minimal voltage fluctuation. Compared with the recently published data, the HER||OER system assembled with CoFePBA-N exhibits comparable performance (Table S2). Even in an alkaline natural seawater environment, the dual-electrode system continued to operate stably at 500 mA cm^−2^ for over 270 h, with extremely small voltage fluctuations (Figure 6i,j), and the actual retention percentages of the catalyst in different electrolytes are 92.1% and 89.3%, respectively. The stability test in seawater is worse than that in fresh water. This is because the Pt@C electrode will severely degrade under the combined effect of the complex seawater environment and high current density (Figure S10). Furthermore, the half-decomposition stability test of Pt@C in alkaline seawater only lasted for about 50 h, which indicates that its poor stability in alkaline seawater limits the overall full decomposition performance (Figure S11). Future work will therefore focus on improving the durability of hydrogen evolution reaction electrodes in alkaline seawater environments. These results demonstrate that the catalyst retains excellent catalytic performance even under complex electrolyte environments.

To survey the structural and chemical state changes in the electrodes after long-term operation, XRD, SEM, and XPS were used to characterize and analyze the tested electrodes. The XRD patterns (Figure S12) show no obvious crystalline phases, indicating the formation of an amorphous reconstruction layer during operation. As shown in Figure S13, SEM observations indicate that the original aggregated framework structure is largely preserved, while the nanosheet-like secondary structure is covered by reconstructed products. The TEM image after electrochemical reconstruction (Figure S14) shows that CoFePBA-N has undergone significant surface reconstruction. The high-resolution transmission image of it does not show any obvious lattice stripes, indicating that its basic reconstruction has become a non-crystalline form of hydroxide. Further XPS analysis (Figure S15) demonstrates that the reconstructed products are still mainly composed of Co, Fe, N, O, and C elements, and the oxidation states of Co and Fe increase after the electrochemical process.

### 2.4. Mechanism for the Enhanced OER Performance

**Figure 7:**
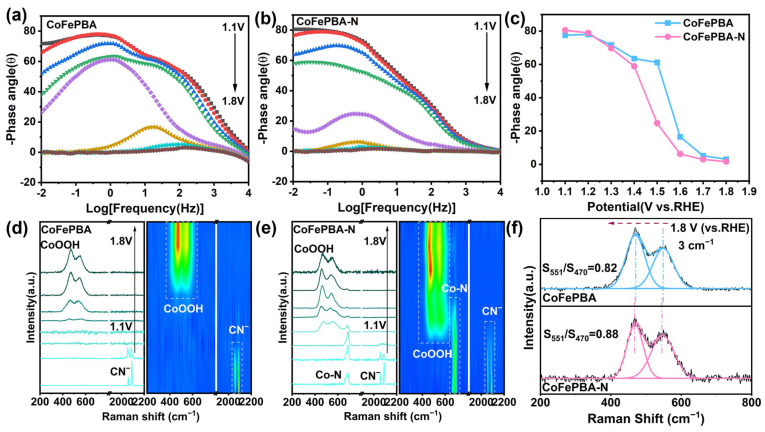
Bode phase plots of CoFePBA (**a**) and CoFePBA-N (**b**). (**c**) The corresponding phase peak angles. In situ Raman spectra and contour maps of (**d**) CoFePBA and (**e**) CoFePBA-N. (**f**) Comparison of in situ Raman spectra at 1.8 V (vs. RHE).

The Bode phase plots illustrate the variation in the phase angle with frequency under different applied potentials. Overall, the peaks in the low-frequency and high-frequency regions are related to the interface charge-transfer process at the catalyst and electrolyte interface, as well as the electron transport process within the electrode layer, respectively [29,30]. For both CoFePBA (Figure 7a) and CoFePBA-N (Figure 7b), the dominant impedance contribution mainly originates from the interfacial charge-transfer process. As shown in Figure 7c, CoFePBA-N consistently exhibits lower phase angles than pristine CoFePBA over the entire potential range. This decrease in phase angle indicates a reduction in interfacial charge-transfer resistance after plasma activation, which is expected to accelerate the kinetics of the OER. To clarify the direct correlation between the catalytic enhancement effect and the dynamic structural evolution, we utilized in situ Raman spectroscopy technology to monitor the potential energy-dependent reconfiguration of the electrode during the oxygen evolution reaction process in real time. As shown in Figure 7d,e, characteristic peaks corresponding to γ-CoOOH and β-CoOOH can be observed for both CoFePBA and CoFePBA-N (470cm^−1^ and 551cm^−1^) [31]. The hydrogen oxygenate ions thus generated through reconfiguration are usually regarded as the active layer that actually performs the catalytic function. With increasing applied potential, the peak intensities continuously increase, confirming that the materials undergo progressive electrochemical oxidation and surface reconstruction. Notably, the CoFePBA-N electrode exhibits the earliest emergence of CoOOH characteristic peaks, and the increase in peak intensity with potential is the most pronounced. This behavior indicates a more extensive and continuous reconstruction of the hydroxide layer. These results clearly demonstrate that plasma modification effectively promotes the early onset of surface reconstruction, enabling the OER to be initiated at lower overpotentials. Such earlier reconstruction represents a key structural origin for the enhanced OER activity. Furthermore, comparative analysis reveals subtle yet significant structural evolution in the reconstructed products of the plasma-modified electrode. As shown in Figure 7f, the CoOOH characteristic peak collected at 1.8 V for CoFePBA-N exhibits a shift of approximately 3 cm^−1^ relative to that of CoFePBA. This shift in the Raman band position generally reflects variations in bond strength and local lattice strain or stress. The observed shift suggests that the Co-O bonds in the reconstructed CoFePBA-N derivatives undergo elongation, accompanied by significant strain relaxation [32]. In CoOOH, such bond elongation is typically associated with enhanced structural flexibility, which facilitates the revelation of active sites and promotes the adsorption energy of the OER intermediate product, thereby significantly improving catalytic activity [33]. Further inspection reveals notable changes in the characteristic peak intensities of γ-CoOOH and β-CoOOH (denoted as S_470_ and S_551_, respectively, in Figure 7f). The S_551_/S_470_ ratio, which is considered a spectroscopic indicator of lattice disorder in CoOOH, increases from 0.82 for CoFePBA to 0.88 for CoFePBA-N. This ratio is positively correlated with the degree of disorder in the crystal structure, indicating that the combined effects of bond angle distortion and vacancy-related perturbations lead to the gradual loss of translational symmetry [34,35]. Overall, these in situ Raman results demonstrate that plasma-induced nitrogen doping and vacancy formation not only accelerate the reconstruction kinetics of CoFePBA-N but also lead to the formation of a more disordered and defect-rich CoOOH surface layer.

## 3. Conclusions

In summary, a nitrogen-doped CoFe Prussian blue analog electrocatalyst was successfully constructed through a plasma-enhanced nitridation strategy. The plasma treatment induces a structural transformation from smooth cubic particles to nanosheet-assembled cubes while simultaneously incorporating nitrogen atoms and vacancies into the framework. This dual regulation effectively optimizes the electronic structure of the Co and Fe active centers and significantly increases the number of exposed catalytic sites. Therefore, the optimized CoFePBA-N exhibits outstanding oxygen evolution reaction performance, with a current density of 500 mA cm^−2^ in alkaline freshwater, an overpotential as low as 322 mV (in alkaline freshwater), 344 mV (in alkaline simulated seawater), and 374 mV (in alkaline seawater), while maintaining stability for over 200 h. When used as the catalyst configuration in the CoFePBA-N||Pt@C electrolytic cell, this system requires a battery voltage of only 1.98 V and 2.08 V in alkaline freshwater and seawater, respectively, and can achieve a current density of 500 mA cm^−2^, and can operate continuously for 200 h. This study highlights the effectiveness of plasma-assisted manufacturing defect technology in adjusting the electronic structure and catalytic performance of Prussian blue-type catalysts, which is a highly promising tactic, offering novel foresight into the rational design of high-performance catalysts for sustainable hydrogen generation.

## Data Availability

The original contributions presented in this study are included in the article and the Supplementary Material. Further inquiries can be directed to the corresponding author.

## Supplementary Materials

The following supporting information can be downloaded at: https://www.mdpi.com/article/10.3390/ma19081580/s1, Figure S1: The sample morphologies of NF (a), CoFePBA (b) and CoFePBA-N (c). Figure S2: The elemental EDX spectra of CoFePBA (a) and CoFePBA-N (b), as well as the elemental distribution map of CoFePBA-N (c). Figure S3: C 1s of CoFePBA and CoFePBA-N. Figure S4: CV curves of CoFePBA-N (a), CoFePBA (b) and RuO_2_ (c) samples at different scanning rates in 1 M KOH. Figure S5: Multiple electrochemical OER tests were conducted on CoFePBA-N. Figure S6. ECSA-normalized LSV curves of CoFePBA, CoFePBA-N and RuO_2_. Figure S7: TOF curves derived from LSV curves of different catalysts in 1 M KOH. Figure S8: CV curves of CoFePBA-N at different scanning rates in 1 M KOH+0.5 M NaCl (a) and 1 M KOH+Seawater (b). Figure S9: (a) The PH of the solution after circulation. (b) The chlorine evolution and color development reaction after the cycle. Figure S10: Pt@C electrode after conducting stability tests in alkaline seawater. Figure S11: The stability test of Pt@C in alkaline seawater. Figure S12: XRD spectrum of CoFePBA-N after electrochemical tests in alkaline seawater solution. Figure S13: SEM images of the CoFePBA-N catalyst after testing its stability for 200 h in 1.0 M KOH+ seawater. Figure S14: TEM images (a) and HR-TEM images (b) of CoFePBA-N-After OER. Figure S15: High-resolution XPS spectra of CoFePBA-N-After OER. (a) Overall survey of pristine, (b) Co 2p, (c) Fe 2p, (d) N 1s, (e) O 1s and (f) C 1s. Table S1: Comparison of the electrocatalytic activity for OER of CoFePBA-N catalyst in 1 M KOH with reported OER catalysts recently. Table S2: Comparison of overall alkaline water splitting performance of CoFePBA-N with other catalysts reported recently. Video S1: The entire seawater decomposition device driven by natural sunlight. During the hydrogen electrode reaction process, severe detachment occurred at the Pt@C. References [36,37,38,39,40,41,42,43,44,45,46,47,48,49] are cited in the supplementary materials.
