# Supplementary material for: Modification of CoFe Prussian Blue Structure by N2 Plasma for Enhanced Electrocatalysis
Source: Materials (Basel). 2026 Apr 15;19(8):1580. doi: 10.3390/ma19081580 (PMC13117872; doi:10.3390/ma19081580)
Supplement: Supplementary file 1 [file materials-19-01580-s001.zip › materials-4226996-supplementary-20260409.pdf]

Supporting Information

1. Experiment

1.1 Chemicals Used:

Ni Foam (AR, 99%), Ethanol (95%, AR). Deionized water (DI water). $CoCl_2 \cdot 6H_2O$, (99%, Macklin), Polyvinylpyrrolidone, PVP (K-17, MW: 10000), Sodium citrate, dihydrate (99%, Macklin), and $K_3[Fe(CN)_6]$) (99%, Macklin), RuO_2, (99.9%, Adamas), KOH (99%, Macklin), argon gas (Ar, >99%), hydrogen gas (H_2, >99%), Nitrogen gas (N_2, >99%).

1.2 Synthesis of CoFe PBA

A nickel foam (NF) substrate (1 cm × 2 cm) was ultrasonically cleaned in hydrochloric acid (HCl), ethanol, and ultrapure water for 5 minutes each, followed by drying. To prepare CoFe PBA, 1.8mmol of $CoCl_2 \cdot 6H_2O$ and $C_6H_5Na_3O_3 \cdot 2H_2O$ (sodium citrate) at a molar ratio of 1:2 were dissolved in 20mL of an aqueous polyvinylpyrrolidone (PVP) solution (1 mg/mL) under continuous stirring, denoted as Solution A. Separately, 40mL of a 0.1 mol/L aqueous $K_3[Fe(CN)_6]$ solution was prepared, and 0.02 g of PVP was added and fully dissolved, denoted as Solution B. Solution A was then gradually added dropwise into Solution B, and the cleaned NF substrate was immersed into the mixture. The system was allowed to stand at room temperature for 24h. The resulting CoFePBA-coated NF was rinsed thoroughly with ultrapure water and dried for subsequent use.

1.3 Synthesis of P-CoFe PBA

The plasma treatment process is carried out using a plasma-enhanced chemical vapor deposition (PECVD) system. The sample is placed in a porcelain boat, which is located within the temperature control zone of the tubular furnace. Under a mixed $Ar/H_2/N_2$ flow (70/2/10 sccm), the temperature was raised to 250°C, and plasma treatment was conducted at 300W for 30 min. After natural cooling to room temperature under the gas flow, the obtained CoFePBA-N was directly employed as the working

electrode.

1.4 Material characterization

The products are characterized by X-ray diffraction (XRD, Rigaku Ultima IV X-ray diffractometer with Cu Kα). Scanning electron micro-scopy (SEM, JEOL Model JSM-78OOF) and high-resolution transmission electron microscopy (TEM, JEOL Model JEM-2100 Plus). Raman spectroscopy (Renishaw in Via Plus with laser at 532 nm). In-situ Raman (Renishaw in Via Plus with laser at 785 nm). In situ Raman cell (C031-3 (3H Gas diffusion type)). The Fourier Transform Infrared Spectroscopy was tested using the FTIR-7600 model instrument. Elemental binding energy measurements were carried out by X-ray photoelectron spectroscopy (XPS, PHI5000 Versaprobe III). The excitation source was monochromatized Al Kα radiation (hν = 1486.6 eV) operated at 25 W. The size of analyzed area was 100 μm (spot diameter) and the electron emission angle was 90°. The sample was tested directly without sputter-etched. During spectra acquisition, the base pressure was 1×10^{-8} Pa. Charge neutralizer was used. Ultraviolet photoelectron spectroscopy measurements were performed using a PHI 5000 VersaProbe III at a base pressure of $1\text{x}10^{-7}$ Torr with He Ia radiation (hν = 21.22 eV) and 2.6 eV of pass energy resolution. The OES spectrum was measured using the AvaSpec-ULS4096CL-EVO model instrument. EPR spectra were obtained using a Bruker A300 spectrometer working in the X band and calibrated with a DPPH (2,2-Diphenyl-1-picrylhydrazyl radical) standard.

1.5 Electrochemical measurements

All electrochemical measurements of the samples are performed at room temperature, and the synthesized catalyst can be directly employed for OER performance evaluation without requiring any post-treatment. The ratio of commercia RuO_2: 4 mg RuO_2 + 15 uL nafion + 135 uL isopropyl alcohol + 50 uL anhydrous ethanol, which was directly applied to 1×2 cm NF. The data is recorded using Gamry electrochemical workstation in a standard three-electrode setup. The reference electrode is a mercuric oxide electrode (Hg/HgO) and the counter electrode is a

Platinum electrode. All the potentials reported in our work are converted to the reversible hydrogen electrode (RHE) scale. The current density in this paper was the apparent current density based on the geometric area of the electrode. Linear scanning voltammetry (LSV) measurements were carried out at a scan rate of 5 mV s^{-1} in 1.0 M KOH、1.0 M KOH + 0.5 M NaCl、1.0 M KOH + Seawater. The potentials presented in this work were referenced to the reversible hydrogen electrode (RHE) through RHE calibration, E(RHE) = E(Hg/HgO) + 0.098 V + (0.0591 × pH) V. The Tafel slopes were calculated by plotting the overpotential against log |j| from LSV data. Before the LSV measurements, the catalysts were measured after 50 cycles of cyclic voltammetry (CV) to reaching a stable state. All LSV curves were corrected by the 85% iR drop compensation. The electrochemical active surface area (ECSA) value was calculated by ECSA = C_{dl}/Cs. Usually, catalysts that have a real surface area of 1 cm^{-2} generally have a specific capacitance value (Cs) in the range of 20-60 μF cm^{-2}. Herein, we took Cs = 40 μF cm^{-2}. The turnover frequency (TOF) is evaluated by the following equation: $TOF = \frac{i*N_A}{4*F*MASD}$ where i is current (A), N_A is the Avogadro constant (6.022×1023 mol^{-1}), 4 is the electron transfer number during OER, and F is the Faraday constant (96485 C mol^{-1}). Metal mass-specific active site density can be calculated using the following equations: $MASD = \frac{A*N_A}{n*v*F*n_{MT}}$ where A is the absolute area of CV curves, N_A is the Avogadro number (6.022×1023 mol^{-1}), n is the number of electrons, v is the scan rate (10 mV s^{-1}), F is the Faraday constant (96485 C mol^{-1}), and n_{MT} is the metal amount of substance of the electrocatalyst loading on NF.

The electrochemical impedance spectroscopy (EIS) was collected at the potential corresponded to the current at 10 mA cm^{-2} in a frequency range from 100 kHz to 0.01 Hz. The charge transfer resistance (R_{ct}) was then determined from the diameter of the semicircle in the Nyquist plots. The electrochemically active surface areas of the catalysts are compared on a relative scale using the capacitance of the electrochemical double layer (C_{dl}) at the non-Faradaic region. Measuring method: In the potential region of non-Faraday currents, CV curves at different sweep speeds were recorded, and the sweep speed range was 5-100 mV s^{-1}. Stability tests used constant current

chronopotentiometry at a continuous current density of 500 mA cm^{-2}.

A commercial polycrystalline silicon solar panel (10 × 10 cm^2) was illuminated under natural sunlight at noon to generate electricity, which powered CoFe PBA-N||Pt@C electrolysis cell. Two digital multimeters were utilized to independently monitor the voltage and current during operation.

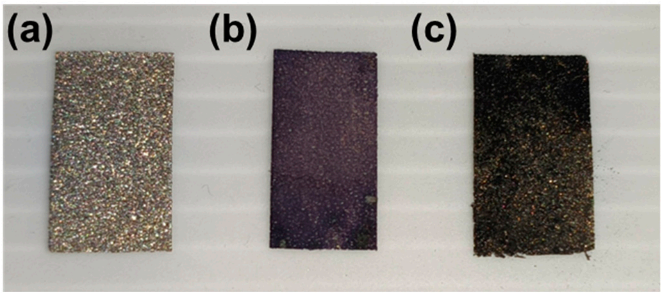

Figure S1. The sample morphologies of NF (a), CoFePBA (b) and CoFePBA-N (c).

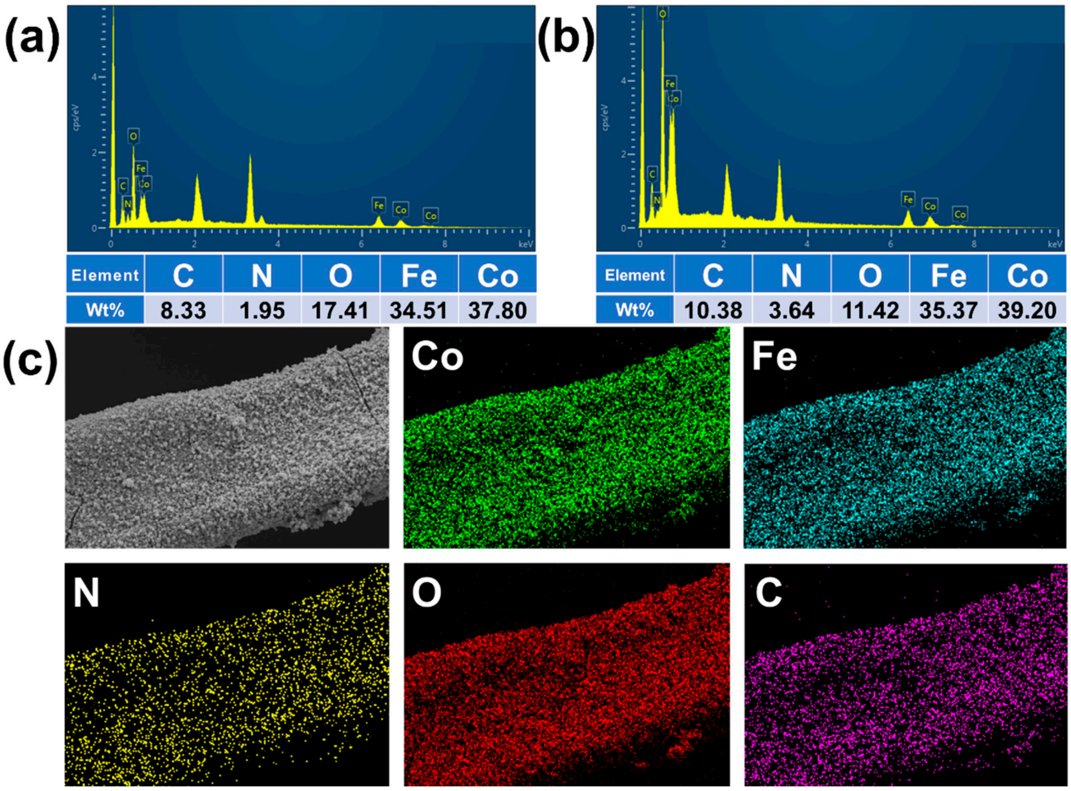

Figure S2. The elemental EDX spectra of CoFePBA (a) and CoFePBA-N (b), as well as the elemental distribution map of CoFePBA-N (c).

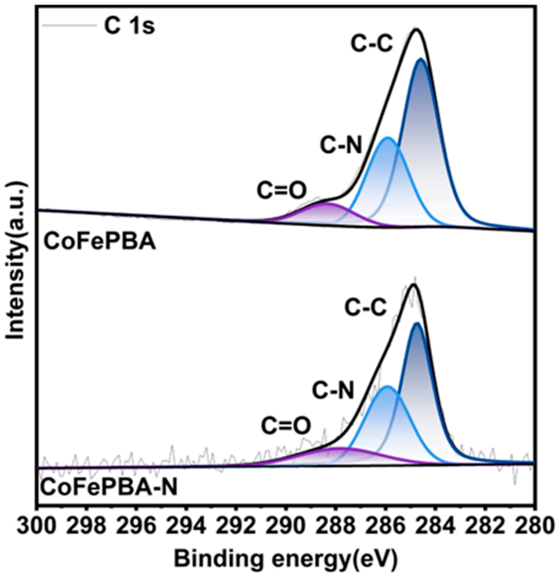

Figure S3. C 1s of CoFePBA and CoFePBA-N.

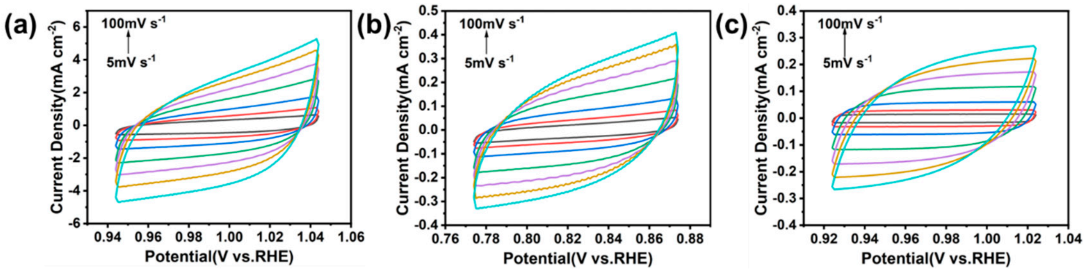

Figure S4. CV curves of CoFePBA-N (a), CoFePBA (b) and RuO_2 (c) samples at different scanning rates in 1 M KOH.

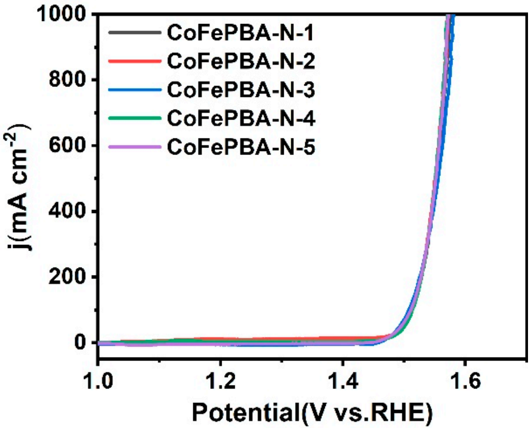

Figure S5. Multiple electrochemical OER tests were conducted on CoFePBA-N

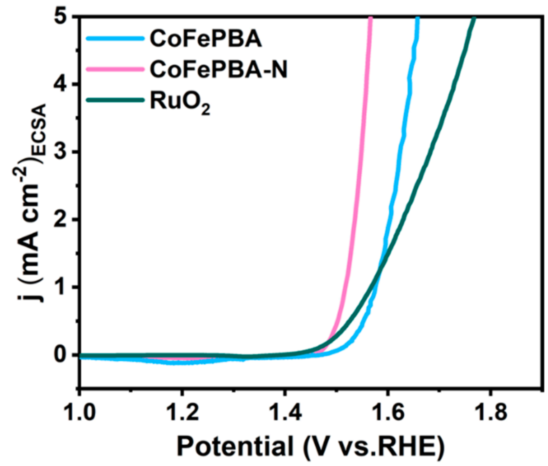

Figure S6. ECSA-normalized LSV curves of CoFePBA, CoFePBA-N and RuO_2.

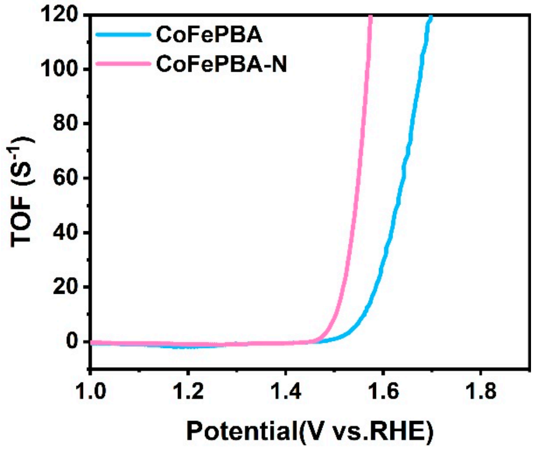

Figure S7. TOF curves derived from LSV curves of different catalysts in 1 M KOH.

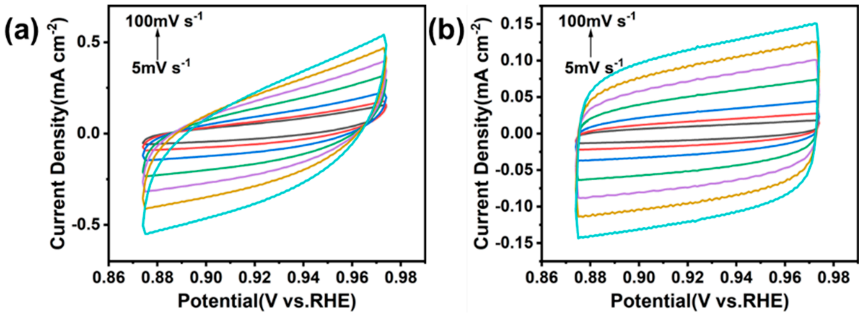

Figure S8. CV curves of CoFePBA-N at different scanning rates in 1 M KOH+0.5 M NaCl (a) and 1M KOH+Seawater (b).

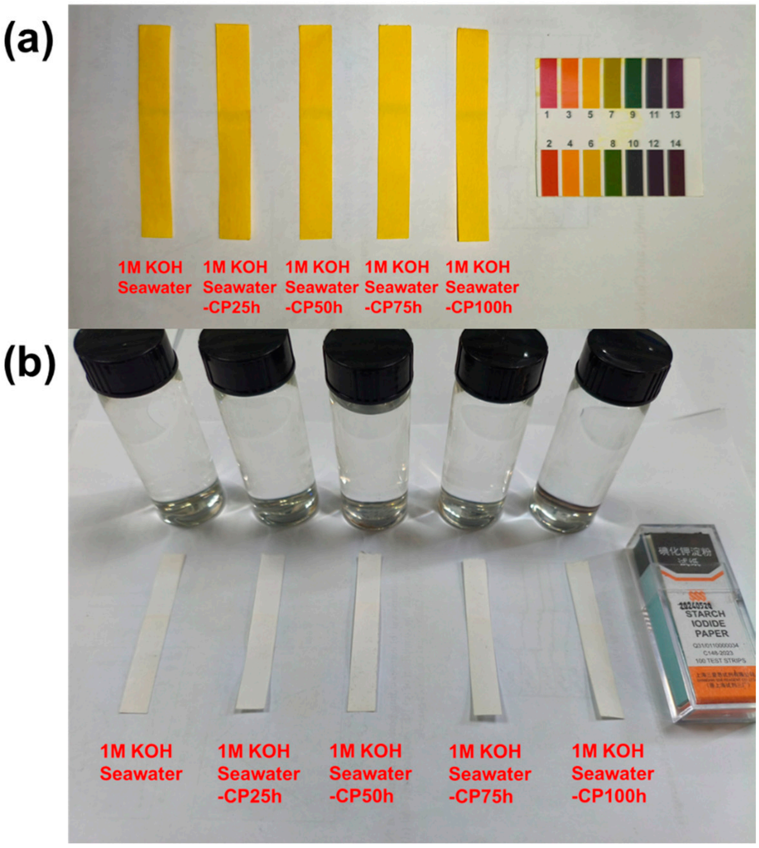

Figure S9. (a) The PH of the solution after circulation. (b) The chlorine evolution and color development reaction after the cycle.

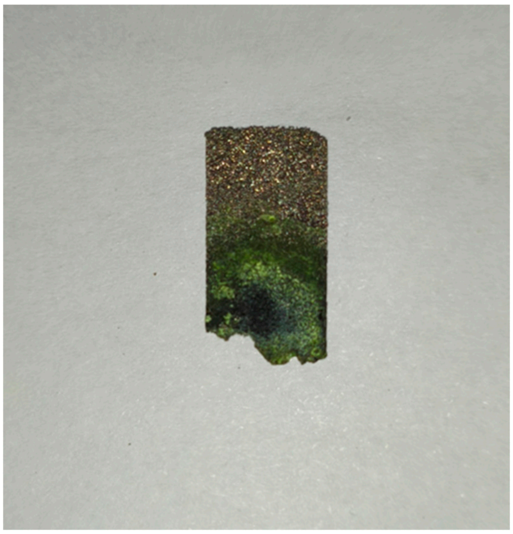

Figure S10. Pt@C electrode after conducting stability tests in alkaline seawater.

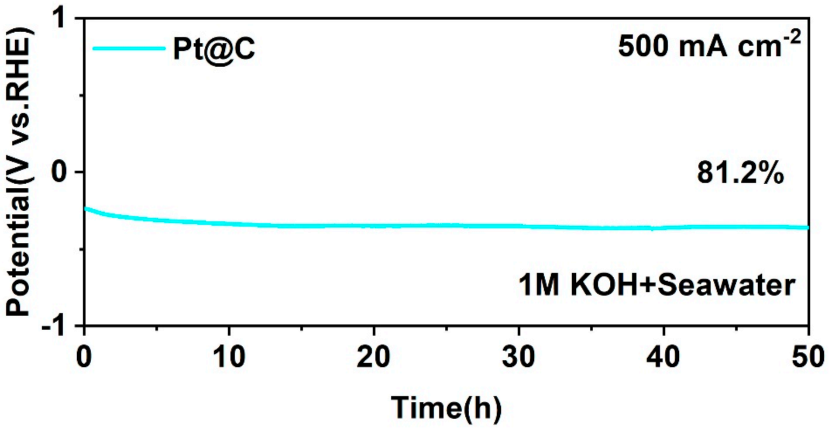

Figure S11. The stability test of Pt@C in alkaline seawater.

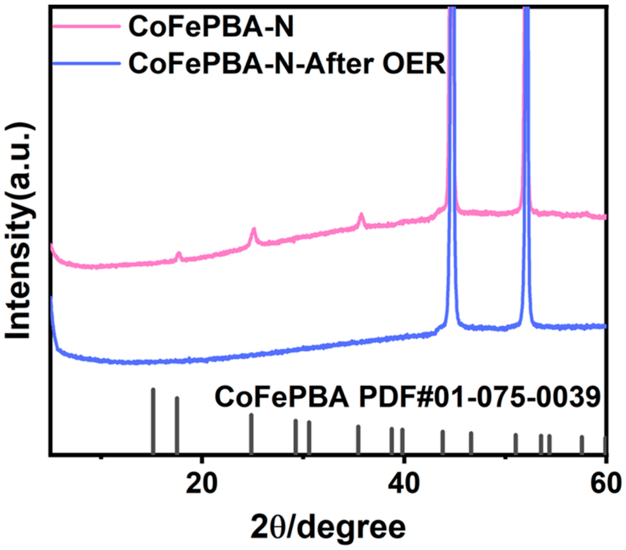

Figure S12. XRD spectrum of CoFePBA-N after electrochemical tests in alkaline seawater solution

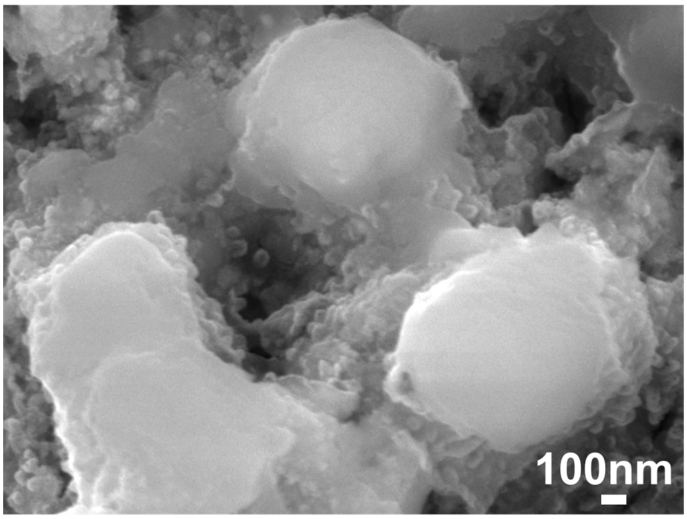

Figure S13. SEM images of the CoFePBA-N catalyst after testing its stability for 200 hours in 1.0 M KOH+ seawater.

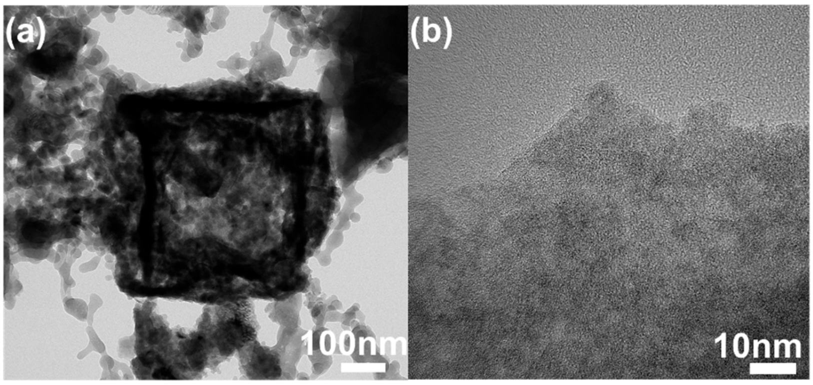

Figure S14. TEM images (a) and HR-TEM images (b) of CoFePBA-N-After OER.

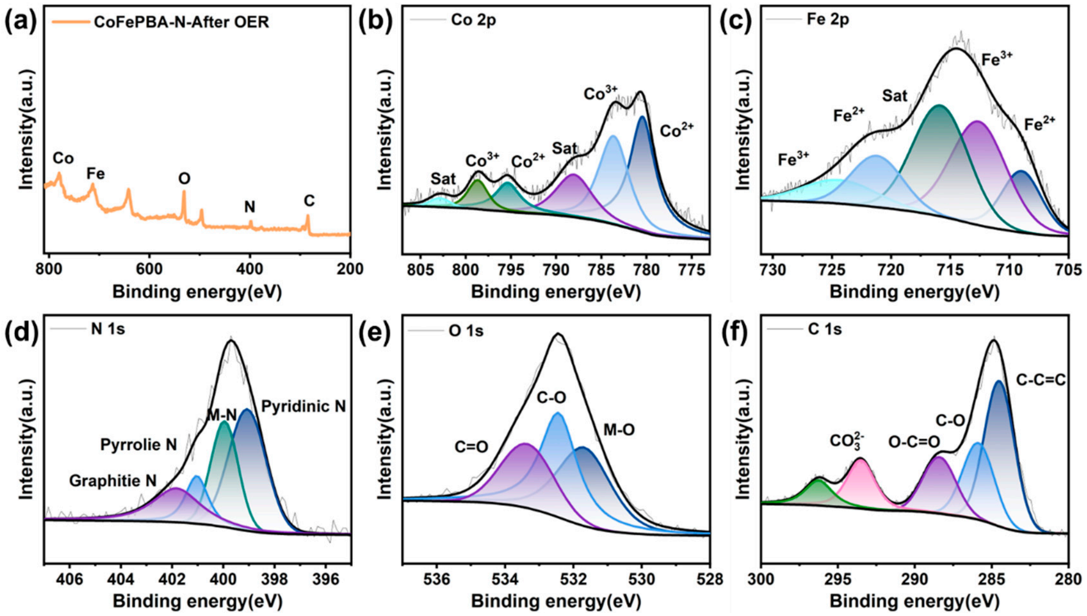

Figure S15. High-resolution XPS spectra of CoFePBA-N-After OER. (a) Overall survey of pristine, (b)Co 2p, (c)Fe 2p, (d)N 1s, (e)O 1s and (f) C 1s.

Video S1. The entire seawater decomposition device driven by natural sunlight. During the hydrogen electrode reaction process, severe detachment occurred at the Pt@C.

Table.S1. Comparison of the electrocatalytic activity for OER of CoFePBA-N catalyst in 1 M KOH with reported OER catalysts recently.

Catalyst	Synthesis method	η (mV) at 500 mA cm^{-2}	Tafel slope (mV dec^{-1})	Ref.
**CoFePBA-N**	**250 °C 30min**	**322**	**38.63**	**This work**
CFP	300 °C 3h	381	29.31	[1]
CoFe/$CoFe_2O_4$@NC	120 °C 96h	456	126.2	[2]
DA-FC-NG	750 °C 2h	353	50	[3]
CFS-Acs/CNT	160 °C 6h	378	77.6	[4]
CoFe/$NC_{30\%}$	180 °C 22h	453	77	[5]
N_3S_4@CoFe-LDH	180 °C 6h	381	70.2	[6]
Vo-CoFe/CoFe2O4@NC	800 °C 3h	468	64	[7]
Ru@CoFe-LDH(3%)	30 °C 30min	352	61	[8]

Table.S2. Comparison of overall alkaline water splitting performance of CoFePBA-N with other catalysts reported recently.

Catalyst	Electrolyte	Voltage	Stable Duratione	Ref.
**CoFePBA-N**	**Fresh water**	**1.98V at 500mA cm^{-2}**	**315h@500 mA cm^{-2}**	**This work**
CoFeZr oxides	Fresh water	1.81V at 350mA cm^{-2}	14h@10mA cm^{-2}	[9]
$CoFe_2O_4$ /CeO_2@C	Fresh water	1.73V at 100mA cm^{-2}	24h@10mA cm^{-2}	[10]
a/c-CoFe-LDH/Pt	Fresh water	1.9V at 100mA cm^{-2}	50h@50mA cm^{-2}	[11]
R-CoFe/Ce/NF	Fresh water	2.0V at 300mA cm^{-2}	100h@300 mA cm^{-2}	[12]
NN-12	Fresh water	2.1V at 500mA cm^{-2}	50h@50mA cm^{-2}	[13]
Ce_{20}/CoFe@C/750	Fresh water	2.1V at 200mA cm^{-2}	24h@300 mA cm^{-2}	[14]
Ce@CoFe-LDH	Fresh water	1.73V at 100mA cm^{-2}	50h@100 mA cm^{-2}	[15]

References

[1] X.R. He, M.H. Liu, F. Liu, X.Z. Liu, H.X. Liao, P.F. Tan, J. Pan, Oxyanion engineering renewable lattice oxygen mechanism of CoFe oxide for enhanced water oxidation, *Adv. Funct. Mater.* **2025** 35 e05936.

[2] F.Y. Guo, J. Luan, X.C. Meng, P. Zheng, W.L. Duan, W.Z. Li, Optimization of conductivity and stability on inverse spinel $CoFe_2O_4$ for alkaline oxygen evolution reaction, *Chem. Eng. J.* **2025** 523 168363.

[3] T.M. Tang, J.Y. Han, Z.L. Wang, X.D. Niu, J.Q. Guan, Diatomic Fe-Co catalysts synergistically catalyze oxygen evolution reaction, *Nano Res.* **2024** 17 3794-3800.

[4] S.R. Xu, S.H. Feng, Y. Yu, D.P. Xue, M.L. Liu, C. Wang, K.Y. Zhao, B.J. Xu, J.N. Zhang, Dual-site segmentally synergistic catalysis mechanism: boosting $CoFeS_x$ nanocluster for sustainable water oxidation, *Nat. Commun.* **2024** 15 1720.

[5] G.A. Gebreslase, M.V. Martinez-Huerta, D. Sebastian, M.J. Lazaro, Transformation of $CoFe_2O_4$ spinel structure into active and robust CoFe alloy/N-doped carbon electrocatalyst for oxygen evolution reaction, *J. Colloid Interface Sci.* **2022** 625 70-82.

[6] A. Karmakar, D. Mahendiran, R. Madhu, P. Murugan, S. Kundu, Bypassing the scaling relationship with spin selectivity: construction of Lewis base-functionalized heterostructural 2D nanosheets for enhanced oxygen evolution reaction, *J. Mater. Chem. A.* **2023** 11 16349-16362.

[7] Y. Go, K. Min, H. An, K. Kim, S.E. Shim, S.H. Baeck, Oxygen-vacancy-rich CoFe/$CoFe_2O_4$ embedded in N-doped hollow carbon spheres as a highly efficient bifunctional electrocatalyst for Zn-air batteries, *Chem. Eng. J.* **2022** 448 137665.
[8] A. Karmakar, R. Jayan, A. Das, A. Kalloorkal, M.M. Islam, S. Kundu, Regulating surface charge by embedding Ru nanoparticles over 2D hydroxides toward water oxidation, *ACS Appl. Mater. Interfaces.* **2023** 15 26928-26938.
[9] L.L. Huang, D.W. Chen, G. Luo, Y.R. Lu, C. Chen, Y.C. Zou, C.L. Dong, Y.F. Li, S.Y. Wang, Zirconium-regulation-induced bifunctionality in 3D cobalt-iron oxide nanosheets for overall water splitting, *Adv. Mater.* **2019** 31 1901439.
[10] Z.L. Hu, C. Wang, F.Q. Li, H.F. Bian, Q. Zhou, G. Xue, S.S. Jia, H. Wu, J. Gu, Y.J. Ma, X.K. Meng, Highly stable bifunctional electrocatalyst based on carbon-supported $CoFe_2O_4$/CeO_2 heterostructure enabled for efficient water splitting, *Chem.-Asian J.* **2026** 21 e00895.
[11] C. Gong, W.X. Li, X. Du, X. He, D.H. Wang, H. Chen, W. Fang, L. Zhao, Y. Chai, Manipulating spin polarization by in-situ reconstructed amorphous/crystalline CoFe-LDH for efficient electrocatalytic water splitting, *Nano Res.* **2025** 18 94907668.
[12] Y. Deng, J. Wang, S.F. Zhang, Z.J. Zhang, J.F. Sun, T.T. Li, J.L. Kang, H. Liu, S. Bai, In situ constructing lamella-heterostructured nanoporous CoFe/$CoFe_2O_4$ and CeO_{2-x} as bifunctional electrocatalyst for high-current-density water splitting, *Rare Met.* **2025** 44 1053-1066.
[13] C.Y.J. Lim, R. Made, Z.H.J. Khoo, C.K. Ng, Y. Bai, J.B. Wang, G.L. Yang, A.D. Handoko, Y.F. Lim, Machine learning-assisted optimization of multi-metal hydroxide electrocatalysts for overall water splitting, *Mater. Horizons.* **2023** 10 5022-5031.
[14] W. Yaseen, K. Harrath, G.Y. Li, B.A. Yusuf, S.C. Meng, M. Xie, I. Khan, J.M. Xie, C.K. Xia, Y.G. Xu, Interface engineering of highly stable CeO_2/CoFe@C electrocatalysts for synergistically boosting overall alkaline water splitting performance, *Inorg. Chem. Front.* **2024** 12 273-290.
[15] X.X. Sun, R.Q. Wang, Q. Wang, K. Ostrikov, Interface-engineered urchin-like CoFe-layered double hydroxide for high-efficiency electrocatalytic oxygen evolution, *Inorg. Chem. Front.* **2024** 11 1458-1471.
